# Construction of Fusion Protein with Carbohydrate-Binding Module and Leaf-Branch Compost Cutinase to Enhance the Degradation Efficiency of Polyethylene Terephthalate

**DOI:** 10.3390/ijms24032780

**Published:** 2023-02-01

**Authors:** Yingxuan Chen, Shudi Zhang, Zhenyu Zhai, Shuo Zhang, Jun Ma, Xiao Liang, Quanshun Li

**Affiliations:** Key Laboratory for Molecular Enzymology and Engineering of Ministry of Education, School of Life Sciences, Jilin University, Changchun 130012, China

**Keywords:** carbohydrate-binding module, poly(ethylene terephthalate), leaf-branch compost cutinase, binding affinity, PET degradation

## Abstract

Poly(ethylene terephthalate) (PET) is a manufactured plastic broadly available, whereas improper disposal of PET waste has become a serious burden on the environment. Leaf-branch compost cutinase (LCC) is one of the most powerful and promising PET hydrolases, and its mutant LCC^ICCG^ shows high catalytic activity and excellent thermal stability. However, low binding affinity with PET has been found to dramatically limit its further industrial application. Herein, *Tr*CBM and *Cf*CBM were rationally selected from the CAZy database to construct fusion proteins with LCC^ICCG^, and mechanistic studies revealed that these two domains could bind with PET favorably via polar amino acids. The optimal temperatures of LCC^ICCG^-*Tr*CBM and *Cf*CBM-LCC^ICCG^ were measured to be 70 and 80 °C, respectively. Moreover, these two fusion proteins exhibited favorable thermal stability, maintaining 53.1% and 48.8% of initial activity after the incubation at 90 °C for 300 min. Compared with LCC^ICCG^, the binding affinity of LCC^ICCG^-*Tr*CBM and *Cf*CBM-LCC^ICCG^ for PET has been improved by 1.4- and 1.3-fold, respectively, and meanwhile their degradation efficiency on PET films was enhanced by 3.7% and 24.2%. Overall, this study demonstrated that the strategy of constructing fusion proteins is practical and prospective to facilitate the enzymatic PET degradation ability.

## 1. Introduction

Poly(ethylene terephthalate) (PET) is a widespread plastic prepared by the polymerization of terephthalic acid (TPA) and ethylene glycol (EG) [1]. Due to its high plasticity, durability and low production cost [2], PET has emerged as a crucial material in the field of construction, packaging and medicine [3], and more than 70 million tons are produced annually worldwide [4]. However, the presence of aromatic groups in the backbone of PET plastics leads to high chemical inertness and environmental stability [5], and the disposal of PET waste has become a serious burden to the environment [6,7]. Currently, the disposal strategies mainly rely on landfilling, incineration, mechanical and chemical recycling [8,9], which are found to possess many limitations such as the occupation of land, emission of harmful gas and dust particles, and lack of practicality [10]. To avoid environmental pollution as well as enable the recycling of PET waste plastics, it is urgent to develop effective strategies for the degradation and recycling of PET materials in an environmentally friendly manner [11].

Biotechnological plastic recycling is to utilize enzymes or engineered microorganisms to hydrolyze PET into monomers for further manufacturing to construct new plastic products [12], which has received recognition due to mild reaction conditions, low energy consumption and environmental friendliness [13]. Moreover, enzymatic PET degradation proceeds in a surface erosion manner, in which the ester bonds of PET are first hydrolyzed to generate the chain ends and further decomposed to produce bis-(2-hydroxyethyl) terephthalate (BHET), mono-(2-hydroxyethyl) terephthalic acid (MHET), TPA and EG [14]. Among the enzymes for PET degradation, leaf-branch compost cutinase (LCC) is the most powerful and promising one owing to its large substrate-binding pocket and suitable thermal stability [15,16], which was discovered using a metagenomic analysis in 2012 [17]. Tournier et al. obtained a thermostable mutant with high catalytic activity, namely LCC^ICCG^, by saturation mutation of residues inside the binding pocket and introduction of disulfide bond by rational design, and the mutant could depolymerize 90% of pretreated PET bottles within 10 h at an industrial level [18]. Despite considerable improvements in catalytic activity and thermal stability [15,18,19], its further industrial application is still hindered by the limited binding ability of LCC^ICCG^ to PET caused by the characteristics of strong hydrophobicity and high crystallinity of PET [20].

During the process of enzymatic degradation, enhancing the binding affinity of enzymes toward the substrates was favorable to improving the effective concentration of enzyme molecules on the substrate surface, thereby obtaining considerable catalytic efficiency [21]. Carbohydrate-binding modules (CBMs) are non-catalytic substrate-binding domains found in cellulases and chitinases that can enhance the binding affinity between enzymes and substrates [22]. CBMs are classified into three types based on the nature of their substrates: type A recognizes the ordered crystalline region of the substrate; type B recognizes the free substrate chain; type C binds the end of the substrate chain [23]. Moreover, type A CBMs can bind the hydrophobic substrates such as cellulose and chitin via the hydrophobic interaction mediated by the conserved aromatic triplets [24]. PET has multiple similar physicochemical properties to cellulose and chitin, including hydrophobicity and high chain density [25,26]. Thus, the fusion proteins consisting of type A CBMs or anchor peptides, and PET hydrolases could improve the binding affinity between enzymes and substrates and further yield ideal PET degradation ability [27,28,29,30].

Herein, the construction of fusion proteins with LCC^ICCG^ and additional auxiliary domains was carried out to improve the binding ability of LCC^ICCG^, in which *Tr*CBM from *Trichoderma reesei* and *Cf*CBM from *Cellulomonas fimi* were rationally selected from CAZy database [31]. First, the PET binding mechanism was investigated using computational biology methods. The fusion proteins were then compared with LCC and LCC^ICCG^ in terms of optimal temperature and pH, thermal stability and pH stability to examine whether the introduction of CBMs altered the enzymatic properties, and meanwhile, the binding affinity of fusion proteins with PET was assessed. Finally, the degradation efficiency of LCC^ICCG^ and fusion proteins on PET films was evaluated using scanning electron microscopy (SEM) and high-performance liquid chromatography (HPLC) regarding PET morphological characteristics and degradation products.

## 2. Results and Discussion

### 2.1. Rational Screening of CBM Domains and Their Binding Mechanism Evaluation

**Figure 1:**
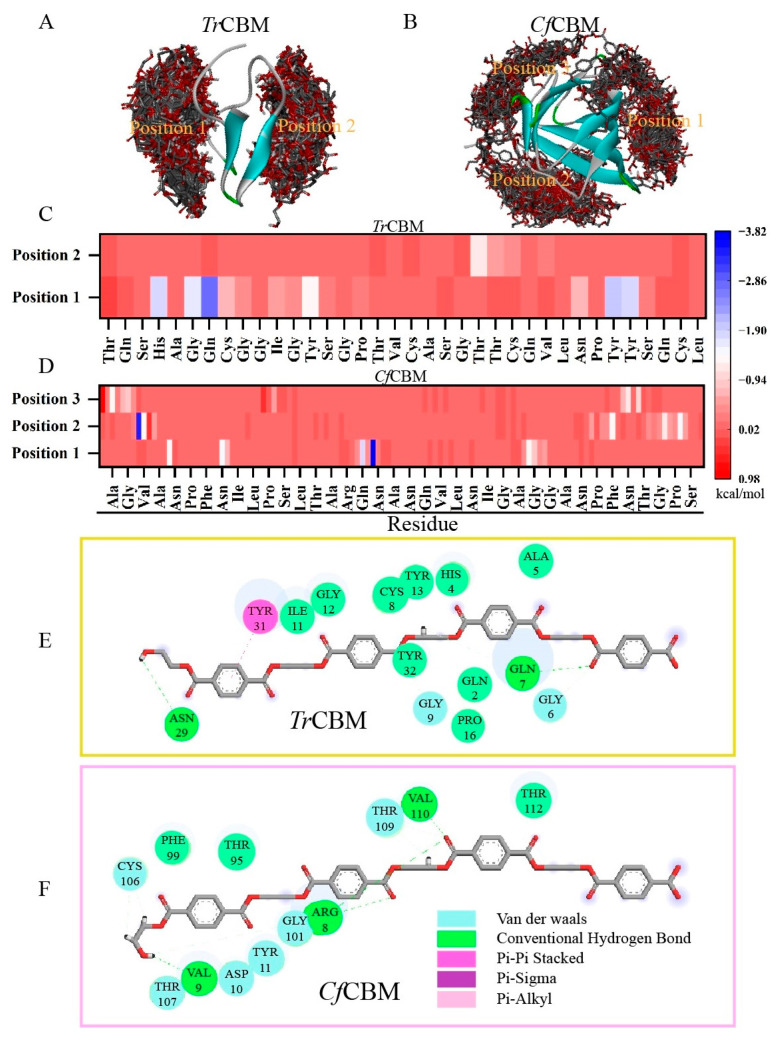
The binding modes of *Tr*CBM (**A**) and *Cf*CBM (**B**) to PET-4 confirmed by blind molecular docking studies using AutoDock Vina software. Molecular graphics were performed using Discovery Studio 4.0. Residue-free energy decomposition of the binding site between *Tr*CBM (**C**) or *Cf*CBM (**D**) and PET-4 in the 500 ns of MD simulation using Amber16. Two-dimensional diagrams for the interaction analysis of *Tr*CBM-Position 1 (**E**) and *Cf*CBM-Position 2 (**F**) with PET-4, in which the gray and red lines represented the carbon and oxygen in the ligand and the circles with different colors represented the main types of force acting on the residues.

First, CBM domains were rationally selected from the CAZy database based on these principles to increase the success rate of selection: (1) CBMs derived from thermophilic microorganisms will be beneficial to adapt the high-temperature conditions for PET degradation; (2) type A CBMs are ideal for binding with the hydrophobic surface of PET owing to the aromatic characteristics in the PET backbone; and (3) the molecular weight of CBMs should be less than LCC^ICCG^. Based on the strategy, *Tr*CBM from *Trichoderma reesei* and *Cf*CBM from *Cellulomonas fimi* were successfully obtained. The structure of *Tr*CBM was acquired from the PDB database (PDB number: 1AZ6), and the catalytic domain of 1AZ6 was removed to gain CBMs for further computational analysis (Figure S1A). As the crystal structure of *Cf*CBM has not been resolved, its structure was constructed using AlphaFold2 prediction [32] (Figure S1B). Before blind molecular docking, the protein structure of *Tr*CBM and *Cf*CBM was optimized, including the hydrogen bond network, protonation state and energy minimization. In addition, the tetramer of 4-((2-hydroxyethoxy) carbonyl) benzoic acid (PET-4) was chosen as the substrate for blind molecular docking, and its structure was constructed and optimized by Avogadro software. Subsequently, blind molecular docking was performed by AutoDock Vina software to analyze the binding mode between CBMs and PET-4. As shown in Figure 1A,B, two and three binding positions of PET-4 were acquired for *Tr*CBM and *Cf*CBM, respectively, and 500 ns of molecular dynamics (MD) simulation was performed to further investigate the free energy and possible interaction mechanism of CBM domains and PET-4. The root mean square deviation (RMSD) results (Figure S2) revealed that the steady state reached after a certain time, indicating the balance of our system. The intercepted trajectory files from the RMSD smoothed area were used for molecular mechanics-generalized born surface area (MM-GBSA) free energy calculations. As shown in Table S1, the binding free energies of *Tr*CBM-Position 1 and 2 were measured to be −31.33 and −3.80 kcal/mol, respectively, and the values of *Cf*CBM-Position 1, 2 and 3 were calculated to be −26.38, −33.38 and −11.21 kcal/mol. All these data suggested that these five positions were favorable for the binding of PET-4 to CBMs in terms of binding energies (the binding energy < 0 kcal/mol) [33]. Among them, *Tr*CBM-Position 1 (−31.33 kcal/mol) and *Cf*CBM-Position 2 (−33.38 kcal/mol) were the most stable binding modes, meaning that these positions were the predominant binding sites of PET-4 to CBMs. In addition, the binding energies of *Cf*CBM-Position 1 and *Cf*CBM-Position 3 also displayed favorable binding activity (the binding energy < −5 kcal/mol) [33], demonstrating that there was more than one site for the binding of substrate PET with *Cf*CBM.

To deeply explore the detailed mechanism, the contribution of single amino acids was evaluated through the calculation of the decomposition of binding energies, and the properties and sources of binding force were also characterized. As shown in Table S2 and Figure 1C, polar amino acids played an important role in the binding process of *Tr*CBM with PET-4, including His4 (−1.84 kcal/mol), Gly6 (−1.72 kcal/mol), Gln7 (−2.83 kcal/mol), Tyr13 (−1.34 kcal/mol), Tyr31 (−1.94 kcal/mol) and Tyr32 (−1.86 kcal/mol). Protein-ligand interaction also revealed that Gln7 was the largest energy contributor, mainly through hydrogen bonding, and Tyr31 executed its contribution via pi–pi interaction (Figure 1E). However, most amino acids played their roles through van der Waals interactions. Similarly, Arg8 (−3.35 kcal/mol), Val9 (−1.28 kcal/mol), Phe99 (−1.34 kcal/mol), Thr109 (−1.20 kcal/mol) and Thr112 (−1.56 kcal/mol) contributed to the binding of *Cf*CBM to PET-4 through van der Waals forces (Table S3), and additionally, hydrogen bonding played a non-negligible contribution (Figure 1D,F). Together, the above results suggested that van der Waals interactions, conventional hydrogen bonding and pi–pi interactions mainly contributed to the binding of CBM to PET-4. In contrast to Joanna’s conclusion [34], the effects of aromatic triplets were not crucial in our system, which was probably caused by the fact that PET-4 used for docking was flexible. Overall, the binding of *Cf*CBM and *Tr*CBM to PET was energetically favorable, suggesting that it potentially improved the binding affinity of LCC^ICCG^ and enhanced the PET degradation through the construction of fusion proteins.

### 2.2. Expression and Purification of LCC, LCC^ICCG^, LCC^ICCG^-TrCBM and CfCBM-LCC^ICCG^

**Figure 2:**
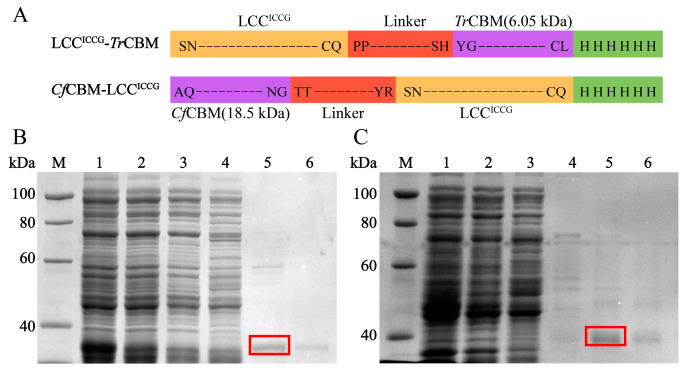
Schematic illustration of the constructed fusion enzymes based on LCC^ICCG^ with CBMs (**A**). SDS-PAGE analysis of LCC^ICCG^-*Tr*CBM (**B**) and *Cf*CBM-LCC^ICCG^ (**C**) expressed in *E. coli* BL21(DE3) during the purification using Ni^2+^-Sepharose 6FF column. Lane M: marker; lane 1: lysates of whole bacterial cells; lane 2: supernatants of lysates; lane 3: effluent fractions of loading sample; lane 4: the fraction eluted with 50 mM imidazole; lane 5: the fraction eluted with 200 mM imidazole; lane 6: the fraction eluted with 500 mM imidazole.

LCC^ICCG^ (Figure S3) was generated through whole plasmid PCR and DpnI digestion using LCC as a template, and *Tr*CBM and *Cf*CBM were fused to LCC^ICCG^ by overlapping PCR (Figure 2A). To avoid the incorrect folding of fusion proteins, *Tr*CBM and *Cf*CBM were fused on the C-terminal or N-terminal of LCC^ICCG^ based on their naturally occurring end, for example, *Tr*CBM at the C-terminus of cellulase [35]. The *Escherichia coli* (*E. coli*) strain BL21(DE3) was transformed with LCC, LCC^ICCG^, LCC^ICCG^-*Tr*CBM and *Cf*CBM-LCC^ICCG^ plasmids and lysed after the IPTG induction. The fusion proteins were purified from the soluble fraction by Ni^2+^-Sepharose 6FF column and then characterized by SDS-PAGE. The theoretical molecular weights of LCC, LCC^ICCG^, LCC^ICCG^-*Tr*CBM and *Cf*CBM-LCC^ICCG^ were 28.5, 28.5, 35.2 and 45.8 kDa, respectively, which matched perfectly with the molecular weights of purified proteins in the SDS-PAGE results, demonstrating the successful expression of fusion proteins (Figure 2B,C and Figure S4A,B). The specific activity of purified enzymes was measured using 4-nitrophenyl caprylate (*p*-NPC) as the substrate, and the catalytic activities of LCC, LCC^ICCG^, LCC^ICCG^-*Tr*CBM and *Cf*CBM-LCC^ICCG^ were calculated to be 18.6, 24.0, 19.3 and 18.7 U/mg, respectively. These results meant that the introduction of CBMs did not significantly influence the catalytic activity of LCC^ICCG^ for the hydrolysis of ester substrates.

### 2.3. Characterization of Fusion Proteins

**Figure 3:**
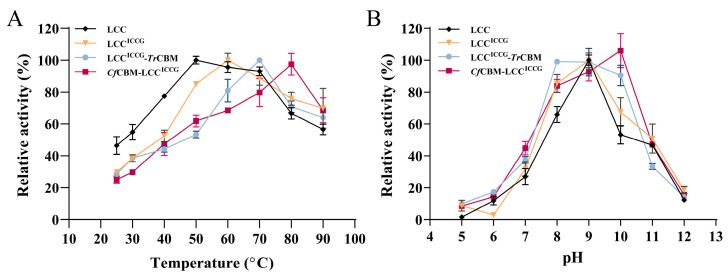
The optimal temperature (**A**) and pH (**B**) of LCC, LCC^ICCG^, LCC^ICCG^-*Tr*CBM and *Cf*CBM-LCC^ICCG^, using the hydrolysis of *p*-NPC as a model. The highest activity was considered as 100%, and relative activities were calculated accordingly. Data were presented as mean value ± SD of triplicate experiments.

To investigate the impact of CBM domains on the enzymatic properties, the optimal temperature and pH were measured using *p*-NPC as a substrate. As shown in Figure 3A, the optimal temperatures for LCC and LCC^ICCG^ were 50 and 60 °C, respectively. Compared to LCC, the optimal temperature of LCC^ICCG^ was elevated by 10 °C, which was caused by the fact that LCC^ICCG^ is a thermally stable mutant of LCC. The result was consistent with the previous findings reported in the literature [18]. Moreover, the optimal temperatures of LCC^ICCG^-*Tr*CBM and *Cf*CBM-LCC^ICCG^ increased to 70 and 80 °C, attributing to the introduction of CBM to enhance the interaction with LCC^ICCG^. Moreover, the optimal pH values of LCC, LCC^ICCG^ and LCC^ICCG^-*Tr*CBM were measured to be 9.0, indicating that the introduction of CBM did not significantly affect the optimal pH. Notably, the optimal pH of *Cf*CBM-LCC^ICCG^ was 10.0, demonstrating its suitable catalytic ability under alkaline conditions.

**Figure 4:**
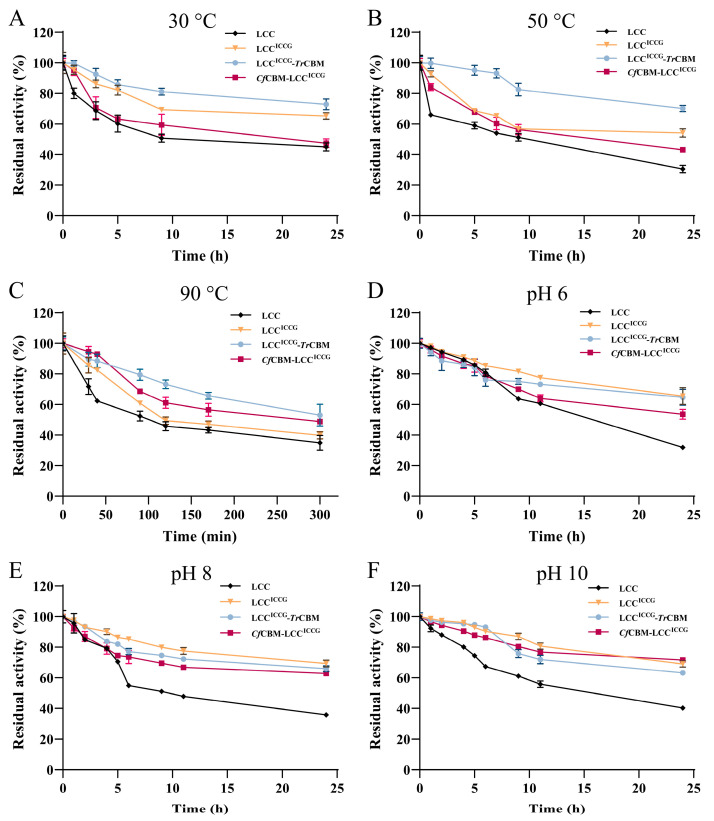
Evaluation of thermal and pH stability using *p*-NPC as a substrate. Residual activity of LCC, LCC^ICCG^, LCC^ICCG^-*Tr*CBM and *Cf*CBM-LCC^ICCG^ after the incubation at 30 °C (**A**), 50 °C (**B**), and 90 °C (**C**) for different times in potassium phosphate buffer (100 mM, pH 8). The residual activity of LCC, LCC^ICCG^, LCC^ICCG^-*Tr*CBM and *Cf*CBM-LCC^ICCG^ after the incubation in Tris-HCl buffer (20 mM) with different pHs of 6 (**D**), pH 8 (**E**) and pH 10 (**F**) at 30 °C for different times, and the activity was monitored in Tris-HCl buffer solution (20 mM, pH 8.0). Data were presented as mean value ± SD of triplicate experiments.

It has been reported that an appropriate reaction temperature and pH can break the hydrophobic interactions within PET, thus favorable for the rapid degradation of PET [18]. However, high-temperature or extreme pH environments can destroy the structure of enzymes, leading to the loss of catalytic activity. The lack of stability is one of the key factors hindering the industrial application of PET hydrolases. To further investigate whether the introduction of CBMs affected the stability of LCC^ICCG^, the temperature and pH stability of fusion proteins were explored. As shown in Figure 4A–C, LCC lost more activity during the initial period and then kept relatively constant with the elongation of incubation time. This phenomenon was probably caused by the aggregation state of LCC, which was consistent with the previous report on the effect of aggregation on enzyme deactivation [36]. When some proteins are unfolded and inactivated, the unfolded enzyme will initiate the aggregation, and aggregated complexes might result in improved stability. However, the microscopic mechanism of this observation is still unknown. LCC^ICCG^-*Tr*CBM maintained 70.1% of initial activity at 50 °C for 24 h, exhibiting the strongest thermal stability, while the residual activities of LCC^ICCG^ and *Cf*CBM-LCC^ICCG^ were 54.2% and 43.2% under the same conditions, respectively. Compared to LCC^ICCG^, the activities of *Cf*CBM-LCC^ICCG^ were relatively lower at 30 °C and 50 °C, implying its decreased thermal stability. The phenomenon was probably caused by the small changes in the structural conformation of fusion protein after the fusion of domain and linker, similar to the previous reports [35,37,38]. Remarkably, LCC^ICCG^-*Tr*CBM and *Cf*CBM-LCC^ICCG^ still retained 53.1% and 48.8% activity after the incubation at 90 °C for 300 min, showing excellent thermal stability. The additional structure could stabilize the catalytic domain of fusion proteins, thereby improving the thermal stability of enzymes [39,40]. Further, thermal inactivation kinetic analysis was conducted via an Arrhenius-type equation to calculate related parameters, including coefficient of thermal inactivation (k_inact_), half-life (t_1/2_) and activation energy (ΔG), as shown in Table S4. Generally, the kinetics of enzyme thermal deactivation is the first order in relation to the concentration of active enzyme, and thus these parameters could be calculated by the Arrhenius law. Clearly, the k_inact_ values of fusion proteins were lower than free enzymes at 90 °C, producing longer half-life values and higher thermostability. Although t_1/2_ of fusion proteins notably increased, the ΔG values of fusion proteins were not significant compared to LCC^ICCG^, with ΔG values of 108.18 and 107.53 kJ/mol for LCC^ICCG^-*Tr*CBM and *Cf*CBM-LCC^ICCG^ at 90 °C, respectively. The results indicated that there was no increase in the inherent conformational stability of protein molecules, but the presence of the fusion module prevented the unfolding of the peptide chain to a certain extent and improved the stability of the protein. In addition, LCC^ICCG^, LCC^ICCG^-*Tr*CBM and *Cf*CBM-LCC^ICCG^ displayed excellent pH stability after the incubation pH 6, 8 and 10 for different times, maintaining more than 60% of initial activity for 24 h (Figure 4D–F). The above findings revealed that both fusion proteins had promising thermal and pH stability, ensuring their potential in the application of PET degradation under complex conditions at an industrial scale.

### 2.4. Adsorption Capacity Analysis of Fusion Proteins on PET Films

**Figure 5:**
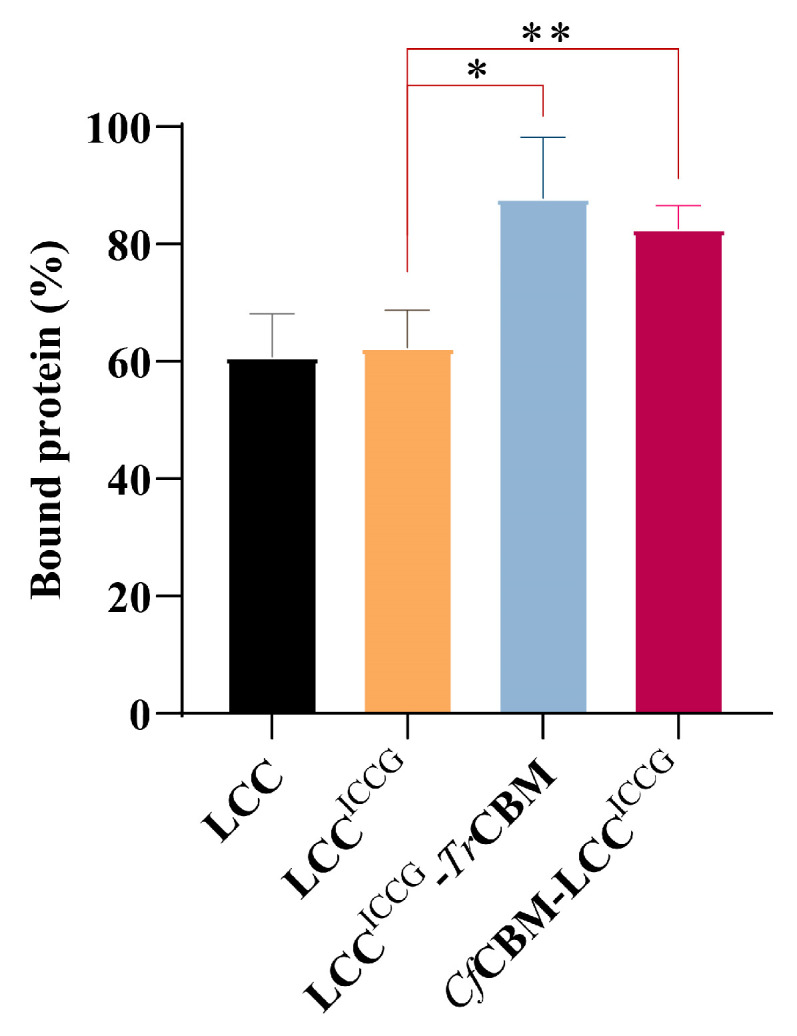
Binding ability analysis of LCC, LCC^ICCG^, LCC^ICCG^-*Tr*CBM and *Cf*CBM-LCC^ICCG^ on PET films. In the experiment, 50 nM of fusion proteins was mixed with 200 μL of potassium phosphate buffer (100 mM, pH 8.0) containing 3 mg of PET films, and the supernatant was collected through centrifugation after the treatment at 4 °C for 24 h. Data were presented as mean value ± SD of triplicate experiments, * *p* < 0.05 and ** *p* < 0.01.

As described above, the thermal stability of fusion proteins was adequate for industrial applications, and thus the binding affinity of fusion proteins to PET was further studied using LCC and LCC^ICCG^ as controls. As shown in Figure 5, the protein adsorption of LCC and LCC^ICCG^ on PET films was almost identical because the four mutant sites did not change the surface charge or hydrophobicity of the enzyme. Excitingly, LCC^ICCG^-*Tr*CBM and *Cf*CBM-LCC^ICCG^ were found to favorably bind onto PET films (up to 87.8% and 82.6% of bound proteins, respectively), improving by 1.4- and 1.3-fold in comparison to LCC^ICCG^. Collectively, the protein adsorption experiments provided direct evidence that the fusion with CBMs was beneficial in enhancing the binding ability of LCC^ICCG^ to PET films. The results were consistent with the computational biology studies, and thus, the construction of fusion proteins could probably facilitate the enzymatic degradation of PET.

### 2.5. Degradation Performance Analysis of Fusion Proteins on PET Films

**Figure 6:**
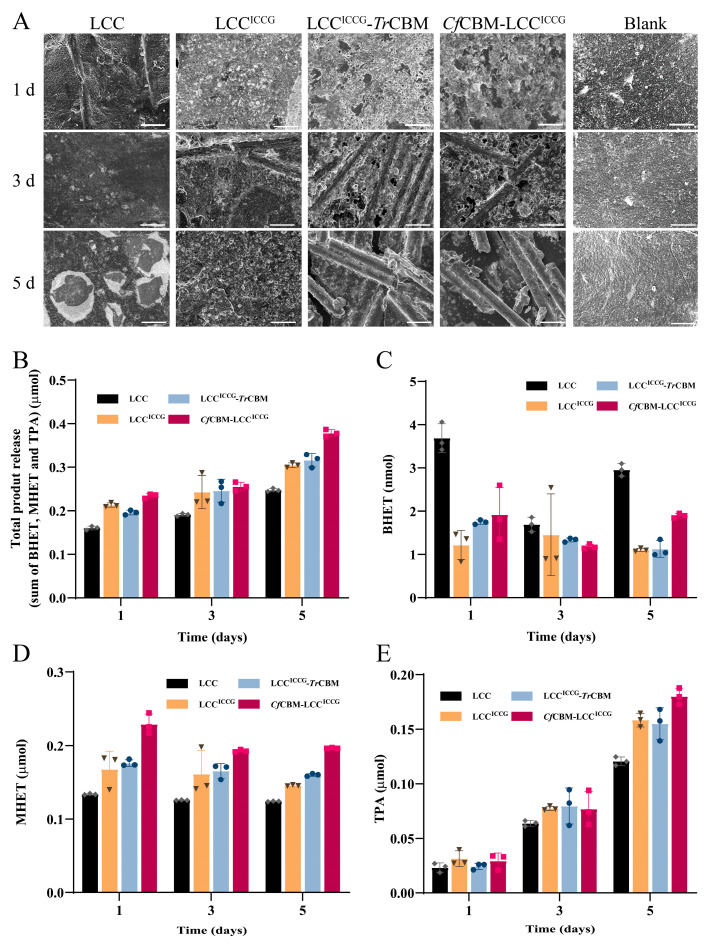
The degradation ability evaluation of fusion proteins on PET films. SEM morphologic analysis of PET films (**A**) recorded before and after enzymatic hydrolysis with LCC, LCC^ICCG^, LCC^ICCG^-*Tr*CBM and *Cf*CBM-LCC^ICCG^ for 1, 3 and 5 days. Scale bar: 50 μm. Release of total degradation products from PET films (**B**), including TPA (**C**), MHET (**D**) and BHET (**E**) after the treatment with LCC, LCCI^CCG^, LCC^ICCG^-*Tr*CBM and *Cf*CBM-LCC^ICCG^ for 1, 3 and 5 days. In the experiment, 0.5 μM of purified enzyme was incubated with 7 mg of PET films in 10 mL of potassium phosphate buffer (100 mM, pH 8.0) at 50 °C and 120 rpm. Data were presented as mean value  ±  SD of triplicate experiments. Rhombus: LCC; inverted triangle: LCC^ICCG^; circle: LCC^ICCG^-*Tr*CBM; square: *Cf*CBM-LCC^ICCG^.

To evaluate the PET degradation capacity of fusion proteins, the degradation reactions of PET films were carried out at 50 °C for a total of 5 days under an environment of pH of 8 using LCC and LCC^ICCG^ as controls, and the content of degradation products and the morphology of PET films were analyzed by HPLC and SEM, respectively. As shown in Figure 6A, the surface of PET films in the blank group presented a smooth and compact morphology and did not show significant structural changes after the treatment under the degradation conditions for 5 days. In contrast, the LCC treatment group showed the erosion of surface structure on day 5, while the LCC^ICCG^ group exhibited stronger degradation ability with numerous erosion sites on day 1 and complete destruction of the surface-dense layer on day 5. Surprisingly, the LCC^ICCG^-*Tr*CBM group performed better degradation efficacy than LCC^ICCG^, with extensive erosion spots on day 1, complete destruction of the surface layer on day 3, and almost complete degradation and exposure of glass fibers on day 5. The 30% glass particles were used as reinforcement in the PET particles. Additionally, the PET degradation of the *Cf*CBM-LCC^ICCG^ treatment group was similar to LCC^ICCG^-*Tr*CBM, revealing that the fusion proteins possessed superior PET degradation capacity than LCC^ICCG^ owing to the improved interaction of enzymes with PET films.

Further, the contents of degradation products, including TPA, MHET and BHET, were examined by HPLC to analyze the degradation efficiency of fusion proteins on PET films. The standard curves were plotted using TPA, MHET and BHET as product standards (Figure S5). The results of total degradation products revealed that the total PET degradation products of all four groups increased with the elongation of incubation time (Figure 6B), indicating that the enzymes were thermally stable enough to maintain their degradation capacity for a long time under the reaction conditions. Moreover, the total products released from the *Cf*CBM-LCC^ICCG^ group kept at the highest level throughout the period, 24.2% higher than LCC^ICCG^, in which the amounts of released BHET, MHET and TPA were 1.97 nmol, 0.20 μmol and 0.18 μmol, respectively (Figure 6C–E). However, the total amount of products of the LCC^ICCG^-*Tr*CBM group was only 3.7% higher than LCC^ICCG^, which did not reflect a significant advantage compared with LCC^ICCG^. The above phenomenon might be attributed to the fact that there was only one binding position between *Tr*CBM and PET, thereby unfavorable for PET degradation mediated by LCC^ICCG^-*Tr*CBM. In contrast, there were three binding positions between *Cf*CBM and PET, which are more beneficial for PET degradation by *Cf*CBM-LCC^ICCG^. Additionally, the LCC group showed the highest release of BHET and exceptionally low release of MHET and TPA. These data also demonstrated the increased degradation capacity or enhanced binding capacity of LCC^ICCG^, LCC^ICCG^-*Tr*CBM and *Cf*CBM-LCC^ICCG^ since MHET and TPA were the products of further hydrolysis of BHET. Different from the characterization of enzymatic properties and binding affinity, the PET degradation of LCC^ICCG^-*Tr*CBM was much weaker than *Cf*CBM-LCC^ICCG^. The unique results were probably caused by the fact that *p*-NPC was used as a model substrate to evaluate the enzymatic properties, not fully reflecting the PET degradation activity. Furthermore, the quantification of total products was not in accordance with the degradation level shown in Figure 6A, which was associated with the degradation pattern of PET. Ren et al. revealed that the degradation of PET was divided into exo- and endo-type degradation [41], and most of the products were poly(4-((2-hydroxyethoxy) carbonyl) benzoic acid)_n_ that could not be detected by HPLC, which was the reason for the discrepancy between SEM and HPLC analysis. The comparison of fusion proteins and other PET degradation enzymes for PET hydrolysis is summarized in Table S5.

The crystallinity was measured before and after enzymatic degradation, which was 8.44% before the degradation and decreased to 6.75% and 5.26% after the degradation with LCC and LCC^ICCG^ for 5 days (Figure S6, Table S6), respectively. Meanwhile, there was no measurable crystallinity in PET films after the treatment with LCC^ICCG^-*Tr*CBM or *Cf*CBM-LCC^ICCG^, indicating that the fusion proteins could significantly improve the degradation ability of the crystalline region of PET materials. Overall, the SEM and HPLC results together elucidated that the PET degradation capacity has been remarkably enhanced for the fusion proteins LCC^ICCG^-*Tr*CBM and *Cf*CBM-LCC^ICCG^.

## 3. Materials and Methods

### 3.1. Materials

The gene encoding leaf-branch compost cutinase (LCC, GenBank: AEV21261), *Tr*CBM (GenBank: AGI55989.1) and *Cf*CBM (GenBank: P07984.1) were synthesized by Sangon Biotech (Shanghai, China) and the gene sequences of fusion proteins were listed in Table S7. *E. coli* BL21(DE3) and DH5α were purchased from TransGen Biotech (Beijing, China). The pET-26b(+) plasmid was obtained from GenScript (Nanjing, China). Ni^2+^-Sepharose 6FF was purchased from Solarbio (Beijing, China). Q5 high-fidelity 2× master mix^®^, DpnI and T4 DNA ligase were purchased from New England Biolabs (Beijing, China). Yeast extract, casein tryptone, agar, kanamycin, ampicillin, PET granules, BHET, MHET and TPA were obtained from Sigma-Aldrich (Shanghai, China).

### 3.2. Rational Screening of CBM Domains and Their Binding Mechanism Evaluation

The structure of *Tr*CBM was derived from protein data bank (PDB: 1AZ6), and the structure of *Cf*CBM was predicted by AlphaFold2 (https://colab.research.google.com/github/sokrypton/ColabFold/blob/main/AlphaFold2.ipynb (accessed on 15 November 2021)) [32]. PET tetramer (PET-4) was selected as the ligand for *Tr*CBM and *Cf*CBM. The molecular docking was conducted through a blind docking approach using AutoDock Vina, with 400 dockings per protein [42].

According to energy scores estimated by AutoDock Vina, two or three binding positions of *Tr*CBM and *Cf*CBM with PET-4 were selected for MD simulation, which was carried out using Amber16 [43]. The charge model AM1-BCC was used to calculate the atomic charges of the ligand. Generalized Amber force field (GAFF) and Amber FF14SB were employed in the analysis of ligand and protein, respectively [44]. Afterward, each protein-ligand complex was immersed in a cubic box with a periodic boundary and a margin of at least 10 Å from any solute atom. The TIP3P water model was used to fill the box for solvating the complex, and the solvated system was neutralized by adding Na ions [45].

The shake algorithm was used to restrain the bonds involving hydrogen atoms in this program, whereas the electrostatic interactions were processed by the Particle-Mesh-Ewald (PME) algorithm with a cut-off of 10 Å [46,47]. To relax the initial structure, an energy minimization scheme was employed with 2500 cycles of steepest descent and 2500 cycles of conjugate gradient minimization. All systems were gradually heated to 300 K during 150,000 steps and equilibrated for 100,000 steps, at which time the step was set to 2 fs in the NTP ensemble. Then, 500 ns (2 fs per step) MD simulation was carried out for each system, and a trajectory was output every 50,000 steps. Subsequently, the RMSD of all MD trajectories was analyzed using the CPPTRAJ program. The MM-GBSA binding free energy between complex and PET-4 was calculated by the MMPBSA.py program, and the obtained binding energies were decomposed to assess the individual energy contributions of the residues in the binding of CBM to PET-4.

### 3.3. Expression and Purification of LCC, LCC^ICCG^, LCC^ICCG^-TrCBM and CfCBM-LCC^ICCG^

The genes of fusion enzymes LCC^ICCG^-*Tr*CBM and *Cf*CBM-LCC^ICCG^ were constructed through overlapping PCR. *E. coli* BL21(DE3) bearing pET-26b(+)-LCC, pET-26b(+)-LCC^ICCG^, pET-26b(+)-LCC^ICCG^-*Tr*CBM and pET-26b(+)-*Cf*CBM-LCC^ICCG^ were cultured at 37 °C for 12 h stirring at 200 rpm. Until OD_600_ reached 0.6, β-D-1-thiogalactopyranoside (IPTG, Sigma-Aldrich, Shanghai, China) was added at a final concentration of 0.5 mM to induce the expression of proteins at 21 °C for 12 h (180 rpm). After that, the cells were harvested by centrifugation at 12,000 rpm for 30 min (4 °C) and disrupted in Tris-HCl buffer (20 mM, pH 8.0) by Ultrasonic Cell Disrupter System (Scientz-II, Ningbo, China). The supernatant was collected by centrifugation at 8000 rpm at 4 °C for 15 min, which was further purified using Ni^2+^-Sepharose 6FF column through 6×His-tag, and the proteins were eluted with 50 mM, 200 mM and 500 mM imidazole, respectively. The eluted fractions containing the target proteins were dialyzed with phosphate buffer (50 mM, pH 8.0), and the protein concentration was determined using bicinchoninic acid (BCA) protein assay kit (SparkJade, Shandong, China). The purified proteins were then characterized using SDS-PAGE analysis in combination with Coomassie brilliant blue (G-250) staining (Dingguo Biotechnol. Co., Beijing, China). The 10% and 12% polyacrylamide gels were prepared by a one-step PAGE gel fast preparation kit (Vazyme, Nanjing, China), and the gel images were captured using a gel imaging system (JUNYI JY04S-3H, Beijing, China).

### 3.4. Characterization of Fusion Proteins

The catalytic activity of LCC, LCC^ICCG^ and fusion proteins were determined using 4-nitrophenyl caprylate (*p*-NPC, Sigma-Aldrich, Shanghai, China) as substrate. In brief, 20 μL of 10 μg/mL protein was added into 980 μL of Tris-HCl (20 mM, pH 8.0) containing 20 μL of 50 mM *p*-NPC, and the change in absorbance at 410 nm was tracked using Shimadzu UV-2700i spectrophotometer (Shanghai, China). One unit of enzymatic activity was defined as the amount of enzyme required to release 1 μmol of *p*-nitrophenol per minute, and the specific activity was calculated by the following equation:

$$\mathrm{Enzyme}\;\mathrm{activity}\;(U/\mathrm{mg})=\frac{\Delta OD410\times V}{\varepsilon 410\times VE\times \left[E\right]\times l}$$

*V*: Total volume of reaction (mL); *ε*410: extinction coefficient of *p*-nitrophenol (M^−1^·cm^−1^); *VE*: volume of enzyme added (mL); [*E*]: enzyme concentration (mg/mL); *l*: light range (cm).

In the optimal temperature and thermal stability, enzymatic activity was analyzed in the range of 25–90 °C using potassium phosphate buffer (100 mM, pH 8.0). In the measurement of optimal pH, proteins were first diluted to 10 μg/mL by different Tris-HCl buffer solutions (20 mM, pH 5.0–12.0), and then the activity was measured in the corresponding buffer solution at 30 °C. In the pH stability test, the enzyme solution (10 μg/mL) was pretreated in Tris-HCl buffer (20 mM, pH 6, 8 or 10) at different times, and the activity was monitored in Tris-HCl buffer solution (20 mM, pH 8.0). All data were obtained from three separate trials. The highest activity was considered as 100%, and relative activities were calculated accordingly. Thermal inactivation kinetics of LCC, LCC^ICCG^ and fusion proteins were conducted based on the data in thermal stability analysis, in which the coefficient of thermal inactivation (k_inact_) and the half-life (t_1/2_) were calculated according to the equations ln(% residual activity) = −k_inact_ × t and t_1/2_ = ln2/k_inact_, and the activation energy (ΔG) was calculated via Arrhenius-type equation as described previously [48]. Each temperature-time combination was performed once, and all data were fitted to a first-order inactivation model.

### 3.5. Adsorption Capacity Analysis of Fusion Proteins on PET Films

PET films were prepared based on the procedure reported in the literature [49]. In brief, 0.1 g of PET granules were dissolved in 1 mL of 1,3,3,3-hexafluoro-2-propanol (Sigma-Aldrich, Shanghai, China), and then the solution was spread flat in a 6 cm glass culture dish (NEST, Wuxi, China). After the evaporation of the solvent, 1 mL of acetonitrile was added dropwise to obtain PET semicrystalline films and dried for 3 days before grinding into fragments in liquid nitrogen. The crystallinity of PET films was measured by differential scanning calorimetry (DSC, Perkin Elmer DSC 7, Norwalk, CT, USA) [41]. DSC was carried out under nitrogen gas purge according to the following procedure: (1) heating from 25 °C to 300 °C at 10 °C/min; (2) holding at 300 °C for 2 min; (3) cooling from 300 °C to 25 °C at 10 °C/min; (4) holding at 25 °C for 2 min; and (5) heating from 25 °C to 300 °C at 10 °C/min. The crystallinity was calculated using the enthalpies of both crystallization and melting based on the following equation [50]:

$$\mathrm{Crystallinity}\;(\%)=\frac{{\Delta H}_{m}-{\Delta H}_{c}}{{\Delta H}_{0}}\times 100\%$$

ΔH*_m_*: Enthalpy of melting change; ΔH*_c_*: enthalpy of crystallization change; ΔH_0_ is the theoretical enthalpy of 100% crystalline PET (ΔH_0_ = 140 J/g).

The adsorption experiments of fusion proteins on PET films were performed as previously described [51]. In brief, 50 nM of purified fusion proteins was added to 200 μL of potassium phosphate buffer (100 mM, pH 8.0) containing 3 mg of PET films. After the incubation at 4 °C for 24 h, the supernatant was collected through centrifugation (13,000 rpm, 1 min). The amount of proteins adsorbed to PET films was assessed by measuring the protein content in the supernatant using a BCA protein assay kit.

### 3.6. Degradation Performance Analysis of Fusion Proteins on PET Films

A total of 0.5 μM of the purified enzyme was incubated with 7 mg of PET films in 10 mL of potassium phosphate buffer (100 mM, pH 8.0) at 50 °C and 120 rpm based on previously described methods [18,51]. The supernatant was collected by centrifugation (13,000 rpm, 5 min) on days 1, 3 and 5, respectively. After boiling at 100 °C for 10 min, the supernatant was collected by centrifugation (10,000 rpm, 2 min) and filtered through a 0.22 μm polyvinylidene fluoride filter (JET BIOFIL, Guangzhou, China). Finally, the samples (10 μL) were analyzed by Shimadzu LC-20 A HPLC system (Shanghai, China) with a C18 column (ShimNex, 4.6 × 250 mm, 5 μm), in which the C18 column was eluted with a solvent (35% methanol, 65% water and 1‰ trifluoroacetic acid, pH 2.5, 1 mL/min) in 0–40 min, monitoring at 254 nm. Besides that, the collected PET films were dried and observed with scanning electron microscopy (SEM, JSM-IT500a, Tokyo, Japan) at an acceleration voltage of 20 kV.

## 4. Conclusions

In conclusion, two CBM domains (*Tr*CBM and *Cf*CBM) with suitable PET binding ability were acquired through rational screening from the CAZy database and then used to construct fusion proteins with LCC^ICCG^ to improve the PET binding and degradation efficiency. Molecular docking and MD simulation revealed that these two domains could bind with PET favorably via polar residues. After the purification of fusion proteins from recombinant *E. coli* BL21(DE3), they were demonstrated to exhibit superior thermal and pH stability and enhanced PET binding ability in comparison to LCC^ICCG^. More importantly, their degradation efficiency on PET films was improved by 3.7% and 24.2% through the quantitative measurement of degradation products. Meanwhile, severely damaged surface morphology could be clearly observed for PET films after the treatment with fusion proteins. These findings provided a useful tool to improve the PET binding and degradation efficiency of PET hydrolases, which was beneficial to achieve the recycling of PET wastes at an industrial scale.

## Data Availability

Data will be made available on request.

## Supplementary Materials

The following supporting information can be downloaded at: https://www.mdpi.com/article/10.3390/ijms24032780/s1. References [52,53,54,55,56,57,58,59,60,61] are cited in the supplementary materials.
