# Supplementary material for: Construction of Fusion Protein with Carbohydrate-Binding Module and Leaf-Branch Compost Cutinase to Enhance the Degradation Efficiency of Polyethylene Terephthalate
Source: Int J Mol Sci. 2023 Feb 1;24(3):2780. doi: 10.3390/ijms24032780 (PMC9917269; doi:10.3390/ijms24032780)
Supplement: Supplementary file 1 [file ijms-24-02780-s001.zip › ijms-2091691-supplementary.pdf]

Supporting Information

Construction of fusion protein with carbohydrate-binding module and leaf-branch compost cutinase to enhance the degradation efficiency of polyethylene terephthalate

Yingxuan Chen[1], Shudi Zhang[1], Zhenyu Zhai, Shuo Zhang, Jun Ma,

Xiao Liang*, Quanshun Li*

Key Laboratory for Molecular Enzymology and Engineering of Ministry of Education, School of Life Sciences, Jilin University, Changchun 130012, China

*Corresponding author.

Tel. and Fax: +86-431-85155200.

E-mail: liang_xiao@jlu.edu.cn (X. Liang); quanshun@jlu.edu.cn (Q. Li).

[1]These authors contributed equally to the work.

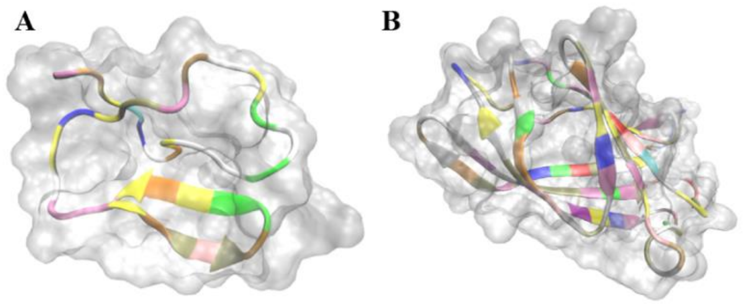

Figure S1. The structure of *Tr*CBM (A) and *Cf*CBM (B) obtained from PDB database (PDB: 1AZ6) or AlphaFold2 prediction, respectively. The display style was based on the amino acids by VMD software.

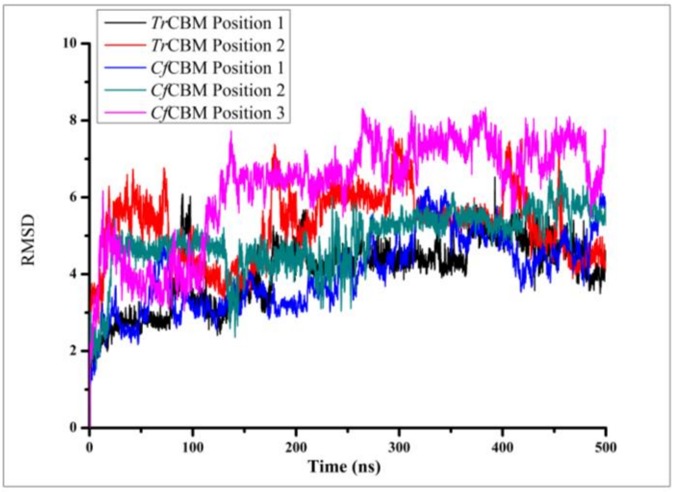

Figure S2. RMSD curves in the 500 ns MD simulation of different positions of *Tr*CBM and *Cf*CBM with PET-4 using Amber16. The charge model AM1-BCC was used to calculate the atomic charges of ligand, and GAFF and Amber FF14SB were employed as force field for the analysis of ligand and protein, respectively.

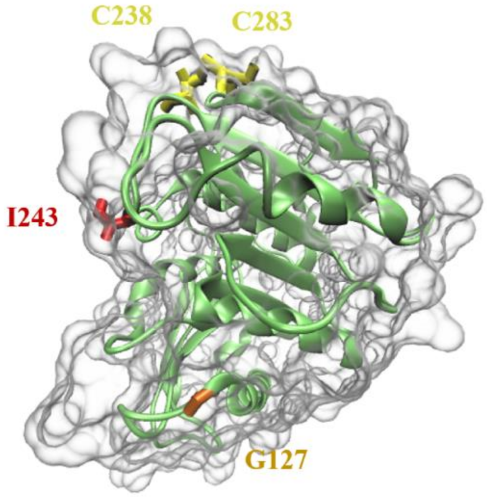

Figure S3. The structure of LCC^{ICCG} obtained from PDB database (PDB: 6THT), in which four mutant locations were highlighted.

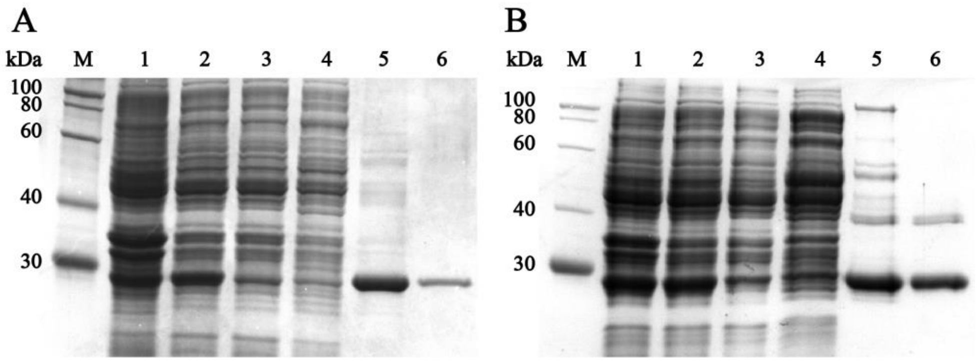

Figure S4. SDS-PAGE analysis of LCC (A) and LCC^{ICCG} (B) expressed in *E. coli* BL21 (DE3). Lane M: protein marker; lane 1: lysates of whole bacterial cells; lane 2: supernatants of lysates; lane 3: effluent fractions of loading sample; lane 4: the fraction eluted with 50 mM imidazole; lane 5: the fraction eluted with 200 mM imidazole; lane 6: the fraction eluted with 500 mM imidazole.

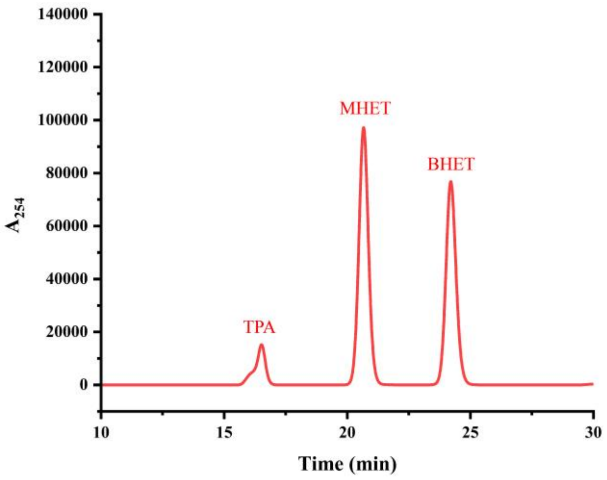

Figure S5. The representative HPLC chromatogram of BHET/MHET/TPA standards. The retention time for these components was 17.4, 21.6 and 23.7 min, respectively.

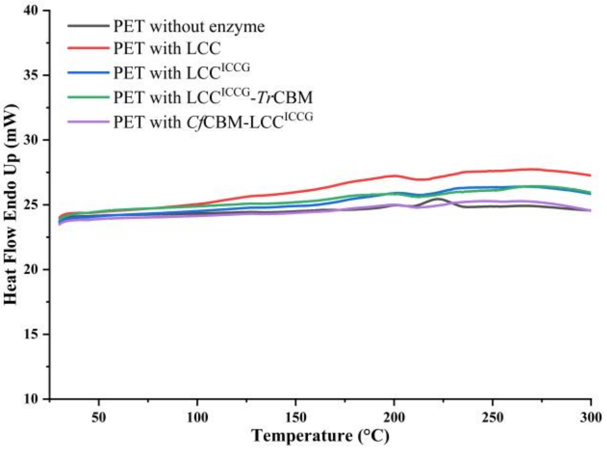

Figure S6. DSC thermograms (2nd heating) of PET films before and after the enzymatic degradation. In the experiment, 0.5 µM of purified enzyme was incubated with 7 mg of PET films in 10 mL of potassium phosphate buffer (100 mM, pH 8.0) at 50 ^{o}C and 120 rpm for 5 days. Black line: PET without enzymatic treatment; red line: PET with LCC treatment; blue line: PET with LCC^{ICCG} treatment; green line: PET with LCC^{ICCG}-*Tr*CBM treatment; and purple line: PET with *Cf*CBM-LCC^{ICCG} treatment.

Table S1. Binding free energy calculation through MM-GBSA (unit: kcal/mol).

System	***Tr*CBM-P1**	***Tr*CBM-P2**	***Cf*CBM-P1**	***Cf*CBM-P2**	***Cf*CBM-P3**
ΔE_{vdw}	-45.46	-7.73	-37.98	-41.44	-22.71
ΔE_{ele}	-17.37	-3.29	-9.10	-33.66	-11.81
ΔG_{GB}	37.19	8.20	25.42	47.45	26.68
ΔG_{gas}	-62.84	-11.02	-47.08	-75.11	-34.53
ΔG_{solv}	31.51	7.23	20.70	41.72	23.32
ΔG_{bind}	-31.33	-3.80	-26.38	-33.38	-11.21

Note: ΔE_{vdw}: van der Waals energy term;

ΔE_{ele}: electrostatic energy term;

ΔG_{GB}: polar solvation energy;

ΔG_{gas}: molecular mechanics term (energy in the gas phase), $\Delta G_{gas} = \Delta E_{vdws} + \Delta E_{ele}$;

ΔG_{solv}: solvation energy, $\Delta G_{solv} = \Delta G_{GB} + \Delta G_{NP}$;

ΔG_{NP}: non-polar solvation energy;

ΔG_{bind}: total free energy of binding, $\Delta G_{bind} = \Delta G_{gas}+\Delta G_{solv}$.

Table S2. Energy breakdown of key residues to *Tr*CBM-Position 1 (unit: kcal/mol).

	System	$\triangle E_{vdw}$	$\triangle E_{ele}$	$\triangle G_{GB}$	$\triangle G_{NP}$	$\triangle G_{gas}$	$\triangle G_{solv}$	$\triangle G_{bind}$
***Tr*CBM-P1**	His4	-2.62	0.15	0.95	-0.32	-2.46	0.62	-1.84
	Gly6	-1.32	-0.78	0.50	-0.12	-2.11	0.38	-1.72
	Gln7	-4.18	-3.86	5.72	-0.49	-8.05	5.22	-2.83
	Tyr13	-1.39	-0.47	0.61	-0.08	-1.87	0.52	-1.34
	Tyr31	-2.20	-0.56	1.19	-0.35	-2.77	0.83	-1.94
	Tyr32	-2.44	0.33	0.58	-0.34	-2.10	0.24	-1.86

Note: ΔE_{vdw}: van der Waals energy term;

ΔE_{ele}: electrostatic energy term;

ΔG_{GB}: polar solvation energy;

ΔG_{NP}: non-polar solvation energy;

ΔG_{gas}: molecular mechanics term (energy in the gas phase), $\Delta G_{gas} = \Delta E_{vdw} + \Delta E_{ele}$;

ΔG_{solv}: solvation energy, $\Delta G_{solv} = \Delta G_{GB} + \Delta G_{NP}$;

ΔG_{bind}: total free energy of binding, $\Delta G_{bind} = \Delta G_{gas}+\Delta G_{solv}$.

Table S3. Energy breakdown of key residues to *Cf*CBM-Position 2 (unit: kcal/mol).

	System	$\triangle E_{vdw}$	$\triangle E_{ele}$	$\triangle G_{GB}$	$\triangle G_{NP}$	$\triangle G_{gas}$	$\triangle G_{solv}$	$\triangle G_{bind}$
	Arg8	-2.94	-9.99	9.99	-0.41	-12.93	9.58	-3.35
	Val9	-1.27	-1.91	2.05	-0.14	-3.18	1.90	-1.28
***Cf*CBM-P2**	Phe99	-1.39	-0.23	0.44	-0.15	-1.63	0.29	-1.34
	Thr109	-1.18	-1.38	1.53	-0.15	-2.57	1.37	-1.20
	Thr112	-1.86	-1.11	1.73	-0.31	-2.98	1.42	-1.56

Note: ΔE_{vdw}: van der Waals energy term;

ΔE_{ele}: electrostatic energy term;

ΔG_{GB}: polar solvation energy;

ΔG_{NP}: non-polar solvation energy;

ΔG_{gas}: molecular mechanics term (energy in the gas phase), $\Delta G_{gas} = \Delta E_{vdw} + \Delta E_{ele}$;

ΔG_{solv}: solvation energy, $\Delta G_{solv} = \Delta G_{GB} + \Delta G_{NP}$;

ΔG_{bind}: total free energy of binding, $\Delta G_{bind} = \Delta G_{gas}+\Delta G_{solv}$.

Table S4. Thermal inactivation kinetic analysis of LCC, LCC^{ICCG} and fusion proteins.

	Temperature (°C)	k_{inact} (h^{-1} or min^{-1})	ΔG (kJ/mol)	$t_{1/2}$ (h or min)
LCC	30	0.02781 h^{-1}	83.33	24.92 h
LCC^{ICCG}	30	0.01684 h^{-1}	84.59	41.16 h
LCC^{ICCG}-*Tr*CBM	30	0.01299 h^{-1}	85.25	53.36 h
*Cf*CBM-LCC^{ICCG}	30	0.02795 h^{-1}	83.32	24.79 h
LCC	50	0.04136 h^{-1}	87.93	16.75 h
LCC^{ICCG}	50	0.02367 h^{-1}	89.43	29.28 h
LCC^{ICCG}-*Tr*CBM	50	0.01544 h^{-1}	90.58	44.89 h
*Cf*CBM-LCC^{ICCG}	50	0.03156 h^{-1}	88.66	21.96 h
LCC	90	0.003133 min^{-1}	106.96	221.24 min
LCC^{ICCG}	90	0.003155 min^{-1}	106.94	219.69 min
LCC^{ICCG}-*Tr*CBM	90	0.002096 min^{-1}	108.18	330.69 min
*Cf*CBM-LCC^{ICCG}	90	0.002595 min^{-1}	107.53	267.10 min

NOTE: The coefficient of thermal inactivation (k_{inact}) and half-life ($t_{1/2}$) values were calculated according to the equations ln(% residual activity) = - $k_{inact} \times t$ and $t_{1/2} = \ln 2/k_{inact}$, and the activation energy (ΔG) was calculated via Arrhenius-type equation.

Table S5. Summary of the activities of PET degrading enzymes and their mutants.

Enzyme	Reference	Type	Mutations	Activity (Substrate)
*Is*PETase	14	Type IIb	WT	+
ThermoPETase	52	Type IIb	S121E/D186H/R280A	14-fold increased activity of S121E/D186H/R280A over WT; 83 μM TA and 37 μM MHET released after 1-10 days
DuraPETase	49	Type IIb	A214H/I168R/W159H/S188Q/R280A /A180I/G165A/Q119Y/L17F/T140D	Over 300-fold enhanced degradation of semi-crystalline (30 %) PET films over WT at 37 °C (semicry-PET)
FAST-PETase	53	Type IIb	ThermoPETase+R224Q/N233K	Depolymerize untreated, amorphous portions of a commercial water bottle and an entire thermally pretreated water bottle at 50 ºC (bottle)
TS-PETase	54	Type IIb	ThermoPETase+N233C/S282C	Increased activity of 6.8-fold after 72 hours; 4.9-fold after 6 days over WT (bottle)
HotPETase	55	Type IIb	TS-PETase+P181V/S207R/S214Y/Q119K/S213E /R90T/Q182M/N212K/R224L/S58A/S61V /K95N/M154G/N241C/K252M/T270Q	At 65 °C, each mole of HotPETase releases 2.7×10^{4} M of monomers in 1 hour, a time-course over which

				reaction progression is linear (cryPET)
LCC	17	Type I	WT	12 mg TA_{eq}×h^{-1}× mg $enzyme^{-1}$ with WT enzyme
LCC-G	19	Type I	N197Q/N266Q/N239G, LCC-G	T_m increased by 10 °C (GF-PET)
LCC^{ICCG}	18	Type I	F243I/D238C/S283C/Y127G (ICCG)	105.6±3.9 mg TA_{eq}×h^{-1}×mg $enzyme^{-1}$, on commercial GF-PET with best varian (GF-PET)
*Cf*CBM-LCC^{ICCG}	This work	Type I	Fusion protein	degradation efficiencies on PET films were enhanced by 24.2% (HPLC) (semicry-PET)
Thc_Cut2	56	Type I	WT	n.d.
Thc_Cut2-2M	57	Type I	G62A/F209A	42% weight loss after 50 h on film G62A/I213S, G62A 2.7-fold better than WT
PET2	58	Type IIa	WT	+
PET2 7 M	59	Type IIa	R47C/G89C/F105R/E110K/S156P/G180A/T297P	6.8-fold increase over WT after 60 min in PET2 7 M variant (GF-PET)
*Fs*C	60	Eukarya	WT	Solubilized 250-μm thick films in 96 h (lc-PET)

*Hi*C	61	Eukarya	WT	+

NOTE: +, activity not quantified; n.d., not determined.

Table S6. Crystallinity of PET films before and after enzymatic degradation.

	**PET film**	**LCC**	**LCC^{ICCG}**	**LCC^{ICCG} -*Tr*CBM**	***Cf*CBM-LCC^{ICCG}**
ΔH_m	11.82	9.44	7.36	0	0
ΔH_c	0	0	0	0	0
Crystallinity	8.44%	6.74%	5.26%	/	/

ΔH_m: Enthalpy of melting change; **ΔH_c:** enthalpy of crystallization change; / meant no measurable crystallinity.

Table S7. The gene sequences of LCC^{ICCG}-*Tr*CBM and *Cf*CBM-LCC^{ICCG}.

LCC^{ICCG}-*Tr*CBM	ATGAGCAACCCGTACCAGCGTGGCCCGAATCCGACC<br>CGCAGCGCACTGACCGCAGATGGCCCGTTTAGCGTGG<br>CAACCTACACCGTCTCACGCCTGTCAGTCTCGGGTTT<br>TGGCGGTGGCGTGATTTATTACCCGACCGGCACGTCT<br>CTGACGTTCGGTGGCATCGCGATGAGTCCGGGTTATA<br>CCGCAGATGCTAGCTCTCTGGCATGGCTGGGTCGTCG<br>CCTGGCTTCCCATGGCTTTGTGGTTCTGGTGATTAACA<br>CGAATTCACGTTTCGATGGCCCGGACAGCCGCGCCTC<br>TCAGCTGAGTGCCGCCCTGAACTACCTGCGTACCAGT<br>TCCCCGAGCGCCGTTCGCGCACGTCTGGATGCAAATC<br>GTCTGGCGGTTGCCGGTCATTCTATGGGTGGCGGTGG<br>CACCCTGCGTATTGCAGAACAAAACCCGAGCCTGAA<br>AGCGGCTGTCCCGCTGACCCCGTGGCACACCGATAAA<br>ACGTTTAATACCAGTGTCCCGGTGCTGATTGTTGGCG<br>CAGAAGCTGACACCGTGGCGCCGGTTTCGCAGCATGC<br>CATCCCGTTTTATCAAAACCTGCCGAGCACCACGCCG<br>AAAGTTTACGTCGAACTGTGCAACGCATCGCACATTG<br>CTCCGAATAGCAACAATGCGGCCATTTCCGTTTATAC<br>GATCTCATGGATGAAACTGTGGGTCGATAATGACACC<br>CGTTACCGCCAGTTCCTGTGTAATGTGAACGACCCGG<br>CTCTGTGCGACTTCCGCACCAATAATCGCCACTGCCA<br>ACCGCCGGGCGGTAACCGTGGCACCACCACCACCCG<br>TCGTCCGGCGACCACCACCGGCAGCTCTCCGGGCCCG<br>ACCCAGAGCCACTACGGCCAGTGCGGCGGCATCGGC<br>TACAGCGGCCCGACCGTTTGCGCGAGCGGCACCACCT<br>GCCAGGTTCTGAACCCGTACTACAGCCAGTGCCTG

*Cf*CBM-LCC^{ICCG}	GCCCAGGCGGCGCCGGGCTGCCGTGTTGATTACGCTG<br>TTACCAACCAGTGGCCGGGCGGTTTCGGCGCGAATGT<br>TACCATCACCAACCTGGGCGATCCGGTTAGCAGCTGG<br>AAACTGGATTGGACCTACACCGCGGGCCAGCGTATCC<br>AGCAGCTGTGGAACGGTACCGCGTCTACCAACGGCG<br>GTCAGGTTAGCGTTACCAGCCTGCCGTGGAACGGCAG<br>CATCCCGACCGGCGGCACCGCGAGCTTCGGCTTCAAC<br>GGTAGCTGGGCGGGTAGCAACCCGACCCCGGCGAGC<br>TTCAGCCTGAACGGTACCACCTGCACCGGCACCGTTC<br>CGACCACCAGCCCGACCCCGACTCCAACCCCGACCAC<br>CCCGACTCCGACCCCGACCCCGACTCCGACTCCGACC<br>CCGACCGTGACCCCGCAGCCGACCTCCGGCTTTTACG<br>TAGATCCGACCACTCAGGGTTACCGTATGAGCAACCC<br>GTACCAGCGTGGCCCGAATCCGACCCGCAGCGCACT<br>GACCGCAGATGGCCCGTTTAGCGTGGCAACCTACACC<br>GTCTCACGCCTGTCAGTCTCGGGTTTTGGCGGTGGCG<br>TGATTTATTACCCGACCGGCACGTCTCTGACGTTCGG<br>TGGCATCGCGATGAGTCCGGGTTATACCGCAGATGCT<br>AGCTCTCTGGCATGGCTGGGTCGTCGCCTGGCTTCCC<br>ATGGCTTTGTGGTTCTGGTGATTAACACGAATTCACG<br>TTTCGATGGCCCGGACAGCCGCGCCTCTCAGCTGAGT<br>GCCGCCCTGAACTACCTGCGTACCAGTTCCCCGAGCG<br>CCGTTCGCGCACGTCTGGATGCAAATCGTCTGGCGGT<br>TGCCGGTCATTCTATGGGTGGCGGTGGCACCCTGCGT<br>ATTGCAGAACAAAACCCGAGCCTGAAAGCGGCTGTC<br>CCGCTGACCCCGTGGCACACCGATAAAACGTTTAATA<br>CCAGTGTCCCGGTGCTGATTGTTGGCGCAGAAGCTGA<br>CACCGTGGCGCCGGTTTCGCAGCATGCCATCCCGTTT<br>TATCAAAACCTGCCGAGCACCACGCCGAAAGTTTACG<br>TCGAACTGTGCAACGCATCGCACATTGCTCCGAATAG<br>CAACAATGCGGCCATTTCCGTTTATACGATCTCATGG<br>ATGAAACTGTGGGTCGATAATGACACCCGTTACCGCC<br>AGTTCCTGTGTAATGTGAACGACCCGGCTCTGTGCGA<br>CTTCCGCACCAATAATCGCCACTGCCAA
